# Global prevalence of poor sleep quality in hemodialysis patients: a systematic review and meta-analysis

**DOI:** 10.3389/fmed.2026.1770352

**Published:** 2026-02-23

**Authors:** Gui-Fen Shi, Xu-Hua Zhou, Lin Chen, Ying-Jun Zhang, Wen-Wen Yu, Jiao Zhang, Li He, Si-Kai Tang

**Affiliations:** 1 Department of Nephrology, Hemodialysis Center, West China Hospital, Sichuan University, Chengdu, Sichuan, China; 2 West China School of Nursing, Sichuan University, Chengdu, Sichuan, China

**Keywords:** epidemiology, hemodialysis, meta-analysis, poor sleep quality, prevalence

## Abstract

**Background:**

Poor sleep quality is associated with various adverse outcomes among hemodialysis (HD) patients. Although poor sleep quality is a widely recognized health issue in HD patients, the reported prevalence in the current literature are remarkably inconsistent. This study aimed to determine the global prevalence of poor sleep quality in HD patients.

**Methods:**

A comprehensive literature search was conducted across seven electronic databases (PubMed, Web of Science, Scopus, Embase, Cochrane Library, CINAHL, PsycINFO) from their inception to October 20, 2025. Data extraction was performed using a standardized form, and the methodological quality of included studies was evaluated with the Joanna Briggs Institute (JBI) critical appraisal checklist for prevalence studies. A random-effects model was applied to calculate the pooled prevalence of poor sleep quality, and the heterogeneity was quantified using the I^2^ statistic. Subgroup analyses and meta-regression were conducted to explore potential sources of heterogeneity.

**Results:**

A total of 69 studies involving 14,998 HD patients were included in the meta-analysis. The pooled global prevalence of poor sleep quality was 64.2% (95% CI: 60.5–67.8%). Based on the JBI critical appraisal tool, 55 studies were rated as having a low risk of bias, while 14 were considered to have a moderate risk of bias. Subgroup analysis revealed that the pooled prevalence varied significantly by the cut-off values. Meta-regression results indicated that prevalence was not significantly associated with sample size, mean age, dialysis duration, and proportion of females.

**Conclusion:**

Our findings demonstrate a high prevalence of poor sleep quality among HD patients. To mitigate the adverse effects of poor sleep quality on HD patients, healthcare providers should routinely conduct screenings and deliver evidence-based interventions.

## 1 Introduction

End-stage renal disease (ESRD) remains a serious global public health challenge (1). Patients with ESRD are dependent on renal replacement therapy to survive, of which hemodialysis (HD) is one of the most common treatments worldwide, supporting the lives of millions of individuals (2). As the number of ESRD patients continues to increase, the primary focus of clinical management has shifted from improving survival rates to enhancing the quality of life for long-term dialysis patients (3).

Sleep quality is generally defined as an individual’s subjective satisfaction with the sleep experience, encompassing sleep initiation, sleep maintenance, sleep depth, and feeling refreshed upon waking, all of which are equally crucial for ensuring a decent quality of life in HD patients (4, 5). Owing to the combined effects of uremic toxin-induced neurological symptoms, dialysis-related circadian rhythm disturbances, and comorbid emotional disorders, all contribute to the prevalent poor sleep quality observed in HD patients (6, 7). It is worth noting that poor sleep quality has a considerable and ongoing effect on the overall health of HD patients (5). Specifically, poor sleep quality is closely associated with numerous clinical adverse outcomes such as dialysis-related fatigue, cardiovascular events, cognitive impairment, infection, and mortality among HD patients (8–11). Therefore, a comprehensive understanding of the prevalence of poor sleep quality in HD patients and its associated factors is crucial for achieving better health outcomes for them.

An accurate estimation of the prevalence of poor sleep quality among HD patients is essential for informing the development of evidence-based prevention and treatment strategies. However, substantial heterogeneity exists in the reported prevalence of poor sleep quality among HD patients across existing studies, with estimates ranging from 31.5 to 93.8% (12–14). Potential resources that contribute to this significant heterogeneity may be attributed to several factors, including different demographic characteristics of the cohorts, instruments used to assess sleep quality, cut-off values, and study designs. To date, there has been no meta-analysis on the global prevalence of poor sleep quality among HD patients. Therefore, this study aimed to determine the global prevalence and its moderating factors of poor sleep quality in HD patients.

## 2 Materials and methods

This meta-analysis adhered to the Preferred Reporting Items for Systematic Reviews and Meta-Analyses (PRISMA) guidelines (15). The protocol was registered with PROSPERO (CRD420251175694).

### 2.1 Search strategy

A comprehensive literature search was conducted across seven electronic databases, including PubMed, Web of Science, Scopus, Embase, Cochrane Library, CINAHL, PsycINFO for articles published from inception until October 20, 2025. The search strategy incorporated MeSH and key terms, including “dialysis,” “hemodialysis,” “hamedialysis,” “sleep quality,” “sleeping quality,” “Pittsburgh sleep quality index,” and “PSQI.” The search syntax for each database is detailed in Supplementary Table S1. Manual searches of the reference lists of included articles and relevant reviews were conducted to identify additional eligible studies.

### 2.2 Inclusion and exclusion criteria

The inclusion criteria were as follows: (1) participants were adult patients (aged ≥ 18 years) on hemodialysis; (2) a cross-sectional or cohort design was adopted (only baseline data included for longitudinal studies); (3) poor sleep quality was defined using a validated instrument such as the PSQI; (4) the prevalence of poor sleep quality was reported or calculable. The exclusion criteria were: (1) reviews, case reports, comments, editorials, or conference abstracts; (2) not published in English; (3) the cut-off values for poor sleep quality were not provided. In cases of duplicate publications, only the study with the largest sample size was retained for analysis.

### 2.3 Data extraction

Data extraction was performed independently by two reviewers using a pilot-tested, standardized Excel form. The following data were extracted: first author, publication year, country, study design, sample size, mean age, dialysis duration, proportion of females, assessment tool, cut-off value, and prevalence of poor sleep quality. All extractions were cross-checked to ensure accuracy, with any discrepancies resolved by consensus.

### 2.4 Risk of bias assessment

The risk of bias in each included study was independently evaluated by two reviewers using the Joanna Briggs Institute (JBI) critical appraisal tool for prevalence studies (16). This checklist includes nine domains, each scored as “yes,” “no,” or “unclear/not applicable.” Based on the proportion of “yes” responses, the overall risk for each study was then classified as low (≥ 70%), moderate (50–69%), or high (≤ 49%). Any discrepancies were referred to a third reviewer and resolved by consensus discussion.

### 2.5 Data analysis

All analyses were conducted using Stata 15.0. The pooled prevalence of poor sleep quality among HD patients and its 95% confidence interval (CI) were derived using a random-effects model. Heterogeneity was evaluated using the I^2^ statistic, with substantial heterogeneity defined as I^2^ ≥ 50% (17). Potential sources of heterogeneity were explored via subgroup analyses based on study design, country income level, and the cut-off values applied. In addition, univariate meta-regression analyses were performed to identify moderators of the prevalence estimates, with sample size, mean age, dialysis duration, and proportion of females serving as covariates. Publication bias was examined visually by inspecting the funnel plot symmetry and statistically using Egger’s test. A leave-one-out sensitivity analysis was undertaken to assess the robustness of the pooled results. *P* < 0.05 was considered statistically significant (two-sided).

## 3 Results

### 3.1 Study selection

**FIGURE 1:**
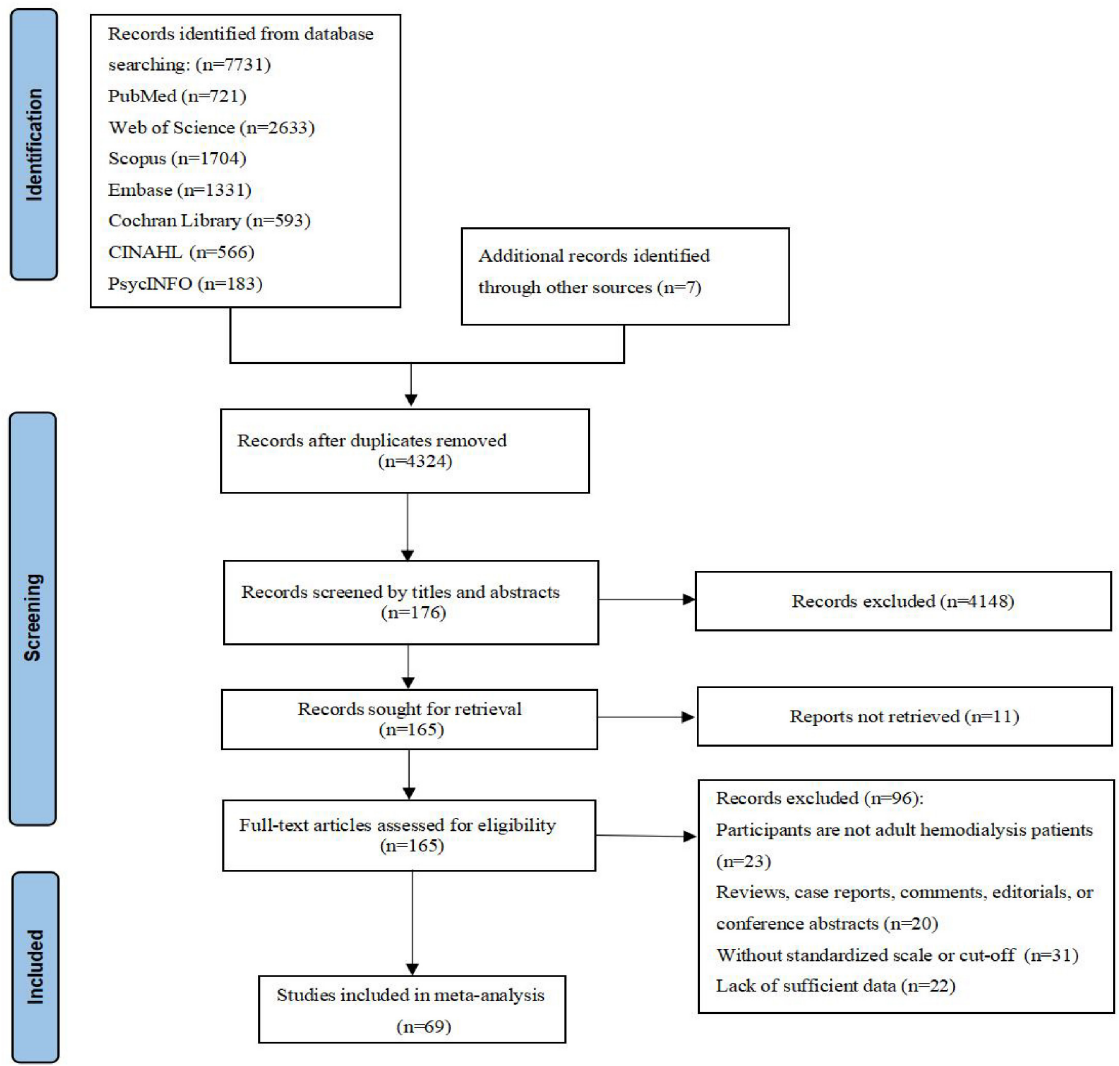
Flow diagram of study selection.

The initial search of electronic databases identified 7,731 records from databases and 7 from manual searches. After the removal of duplicates, 4,324 records were screened based on titles and abstracts. Of these, 165 full-text articles were assessed for eligibility. A total of 69 studies were ultimately incorporated into the meta-analysis. The study screening process is detailed in Figure 1.

### 3.2 Study characteristics

The detailed characteristics of the included studies are shown in Table 1. A total of 14,998 participants from 69 studies (7–11, 14, 18–76) were included, with mean ages ranging from 37.10 to 65.61 years. The included studies were conducted in 19 countries, including Iran (*n* = 11), China (*n* = 10), Turkey (*n* = 10), Pakistan (*n* = 5), Saudi Arabia (*n* = 4), Malaysia (*n* = 4), Somalia (*n* = 3), Canada (*n* = 3), USA (*n* = 3), India (*n* = 3), Brazil (*n* = 3), Palestine (*n* = 2), Korea (*n* = 2), Oman (*n* = 1), Egypt (*n* = 1), Bosnia and Herzegovina (*n* = 1), Italy (*n* = 1), Croatia (*n* = 1), and Serbia (*n* = 1). The sample sizes for individual studies varied from 46 to 1,281. All studies utilized the PSQI to assess sleep quality, employing different cut-off values: a PSQI score ≥ 5 was used in 26 studies, a score ≥ 6 in 41 studies, and a score ≥ 7 in 2 studies. The prevalence of poor sleep quality reported in the included studies varied considerably, with a range of 31.5–93.8%.

**TABLE 1:** Characteristics of included studies.

Study	Country	Study design	Sample size	Mean age (years)	Dialysis duration (years)	Female (%)	Assessment tool	Cut-off value	Prevalence (%)
Abforoushha et al. (18)	Iran	Cross-sectional	150	65.61 ± 4.07	3.37 ± 2.20	49.3	PSQI	≥ 6	54.1%
Al Naamani et al. (6)	Oman	Cross-sectional	123	NR	NR	32.5	PSQI	≥ 5	56.9%
Almutary (7)	Saudi Arabia	Cross-sectional	116	50.66 ± 12.73	NR	54.3	PSQI	≥ 5	56.9%
Alshammari et al. (8)	Saudi Arabia	Cross-sectional	260	NR	NR	42.7	PSQI	≥ 5	37.7%
Anwar and Mahmud (19)	Pakistan	Cross-sectional	113	NR	NR	53.1	PSQI	≥ 5	72.6%
Araujo et al. (20)	Brazil	Cross-sectional	400	51.6 ± 15.5	5.9 ± 5.5	59.0	PSQI	≥ 6	56.7%
Badr et al. (14)	Egypt	Cross-sectional	81	47.2 ± 7.6	NR	48.1	PSQI	≥ 5	93.8%
Bastos et al. (21)	Brazil	Cross-sectional	100	46.1 ± 15.5	5.00 ± 4.58	41.0	PSQI	≥ 6	75.0%
Bilgic et al. (22)	Turkey	Cross-sectional	67	47.7 ± 11.4	8.64 ± 4.93	49.3	PSQI	≥ 6	44.8%
Carneiro et al. (23)	Brazil	Cross-sectional	48	48.32 ± 12.37	5.12 ± 3.82	45.8	PSQI	≥ 6	68.8%
Čengić et al. (24)	Bosnia and Herzegovina	Cross-sectional	200	56.8 ± 14.3	5.22 ± 4.75	39.0	PSQI	≥ 6	73.5%
Choudhary et al. (25)	India	Cross-sectional	66	NR	NR	40.9	PSQI	≥ 6	74.2%
Daraghmeh et al. (26)	Palestine	Cross-sectional	250	54.9 ± 15.08	NR	37.2	PSQI	≥ 5	66.4%
Davison et al. (27)	Canada	Cross-sectional	205	60.0 ± 15.9	2.77 ± 4.12	42.0	PSQI	≥ 7	61.5%
D’Onofrio et al. (28)	Italy	Cross-sectional	103	NR	NR	37.9	PSQI	≥ 5	56.3%
Erickson et al. (29)	USA	Cross-sectional	160	58 ± 14	4.13 ± 4.18	45.0	PSQI	≥ 5	90.6%
Eslami et al. (30)	Iran	Cross-sectional	190	NR	NR	39.5	PSQI	≥ 5	85.8%
Firoz et al. (31)	Iran	Cross-sectional	310	59.64 ± 13.94	NR	47.7	PSQI	≥ 6	73.5%
Gencdal et al. (32)	Turkey	Cross-sectional	137	49.74 ± 12.49	3.45 ± 3.87	38.7	PSQI	≥ 6	63.5%
Han et al. (33)	China	Cross-sectional	141	59.7 ± 15.3	NR	39.0	PSQI	≥ 5	62.4%
Harris et al. (10)	USA	Cohort	128	57.3 ± 13.8	NR	39.8	PSQI	≥ 6	45.3%
Ho et al. (4)	Malaysia	Cross-sectional	184	54.3 ± 12.6	NR	39.1	PSQI	≥ 6	51.1%
Hosseini et al. (13)	Iran	Cross-sectional	175	51.6 ± 16.4	NR	36.4	PSQI	≥ 6	78.2%
Iliescu et al. (34)	Canada	Cross-sectional	89	60.1 ± 16.8	NR	38.2	PSQI	≥ 6	70.8%
Jeele et al. (35)	Somalia	Cross-sectional	299	56.65 ± 12	NR	45.8	PSQI	≥ 6	61.9%
Ji et al. (36)	Korea	Cross-sectional	175	56.9 ± 13.8	5.5 ± 5.5	44.0	PSQI	≥ 6	73.7%
Joshwa et al. (37)	India	Cross-sectional	47	37.1 ± 13.1	NR	49.0	PSQI	≥ 5	68.1%
Kang et al. (38)	Korea	Cross-sectional	101	57.3 ± 12.2	2.98 ± 3.00	45.1	PSQI	≥ 5	75.2%
Kaya et al. (39)	Turkey	Cross-sectional	232	60.9 ± 14.1	3.57 ± 3.63	43.5	PSQI	≥ 6	34.9%
Kir et al. (40)	Turkey	Cross-sectional	338	NR	NR	47.6	PSQI	≥ 6	41.4%
Kose et al. (12)	Somalia	Cross-sectional	200	52.3 ± 14.13	NR	41.5	PSQI	≥ 6	31.5%
Lin et al. (41)	China	Cross-sectional	120	NR	NR	46.7	PSQI	≥ 6	92.5%
Ling et al. (42)	Malaysia	Cross-sectional	184	54.3 ± 12.6	NR	39.1	PSQI	≥ 6	51.1%
Liu et al. (43)	China	Cross-sectional	201	51.1 ± 9.0	NR	44.3	PSQI	≥ 6	43.3%
Maung et al. (44)	USA	Cross-sectional	69	55.6 ± 16.6	NR	50.7	PSQI	≥ 6	58.0%
Mohamed et al. (45)	Somalia	Cross-sectional	200	52.29 ± 14.13	NR	41.5	PSQI	≥ 6	31.5%
Monfared et al. (46)	Iran	Cross-sectional	126	54.9 ± 16.1	NR	38.9	PSQI	≥ 6	44.6%
Mortazavi et al. (47)	Iran	Cross-sectional	160	60.01 ± 13.52	6.37 ± 6.02	33.7	PSQI	≥ 6	84.4%
Morvaridi et al. (48)	Iran	Cross-sectional	423	52.83	4.95	40.7	PSQI	≥ 6	60.5%
Naeem Alharbi (49)	Saudi Arabia	Cross-sectional	100	NR	NR	58.0	PSQI	≥ 6	65.0%
Ng et al. (50)	Malaysia	Cross-sectional	217	57 ± 13	4.62 ± 4.06	50.2	PSQI	≥ 6	54.8%
Norozi Firoz et al. (51)	Iran	Cross-sectional	310	59.64 ± 13.94	3.32 ± 3.55	47.7	PSQI	≥ 6	59.7%
Ongan and Yuksel (52)	Turkey	Cross-sectional	103	59.19 ± 14.57	NR	51.5	PSQI	≥ 5	62.1%
Pai et al. (53)	China	Cross-sectional	164	57.9 ± 11.8	NR	53.0	PSQI	≥ 6	74.4%
Pan et al. (54)	China	Cross-sectional	178	62.9 ± 11.5	4.73 ± 3.35	42.0	PSQI	≥ 6	60.1%
Parvan et al. (55)	Iran	Cross-sectional	245	58.03 ± 14.03	NR	35.5	PSQI	≥ 6	83.3%
Pojatić et al. (56)	Croatia	Cross-sectional	170	NR	NR	40.6	PSQI	≥ 5	68.8%
Ramezanzade et al. (57)	Iran	Cross-sectional	225	58.23 ± 13.50	NR	41.3	PSQI	≥ 5	72.0%
Rehman et al. (58)	Pakistan	Cross-sectional	354	NR	NR	33.9	PSQI	≥ 6	74.0%
Sabbagh et al. (59)	Canada	Cross-sectional	46	61.9 ± 16.9	NR	34.0	PSQI	≥ 5	76.1%
Sabet et al. (60)	Iran	Cross-sectional	61	52.5 ± 18.0	NR	32.8	PSQI	≥ 6	73.8%
Samara et al. (61)	Palestine	Cross-sectional	167	57.6 ± 12.9	NR	47.9	PSQI	≥ 5	76.7%
Shen et al. (62)	China	Cross-sectional	68	61.75 ± 16.56	2.64 ± 2.45	36.8	PSQI	≥ 5	69.1%
Soleimani Damaneh et al. (63)	Iran	Cross-sectional	423	52.84 ± 14.63	4.12 ± 5.12	41.1	PSQI	≥ 6	60.5%
Taraz et al. (64)	Iran	Cross-sectional	72	56.68 ± 15.79	5.93 ± 5.36	41.7	PSQI	≥ 5	75.0%
Terzi et al. (65)	Turkey	Cross-sectional	50	64.46 ± 14.61	NR	56.0	PSQI	≥ 5	82.0%
Tian et al. (9)	China	Cohort	613	63.7 ± 7.8	NR	42.1	PSQI	≥ 5	77.0%
Tian et al. (11)	China	Cohort	595	57.40 ± 13.72	NR	41.2	PSQI	≥ 7	46.7%
Trbojević-Stanković et al. (66)	Serbia	Cross-sectional	222	57.3 ± 11.9	5.12 ± 5.03	40.5	PSQI	≥ 6	64.0%
Türk et al. (67)	Turkey	Cross-sectional	220	NR	NR	50.9	PSQI	≥ 6	51.8%
Uysal et al. (68)	Turkey	Cross-sectional	102	NR	NR	48.0	PSQI	≥ 5	59.8%
Velu et al. (69)	India	Cross-sectional	148	44 ± 14.5	NR	31.8	PSQI	≥ 5	68.2%
Xu et al. (70)	China	Cross-sectional	193	53.09 ± 11.68	NR	34.2	PSQI	≥ 6	63.7%
Yang et al. (71)	China	Cross-sectional	861	NR	NR	55.2	PSQI	≥ 5	83.7%
Yavuz et al. (72)	Turkey	Cross-sectional	121	NR	NR	54.5	PSQI	≥ 6	46.3%
Zhang et al. (73)	China	Cross-sectional	741	60 ± 14	NR	62.1	PSQI	≥ 6	62.2%
Zhang et al. (74)	China	Cross-sectional	1281	54.48 ± 13.09	NR	34.7	PSQI	≥ 6	58.5%
Zubair and Butt (75)	Pakistan	Cross-sectional	140	NR	NR	27.9	PSQI	≥ 5	68.6%
Zubair and Butt (76)	Pakistan	Cross-sectional	137	NR	NR	27.7	PSQI	≥ 5	66.4%

### 3.3 Risk of bias assessment of included studies

The risk of bias assessment results were detailed in Supplementary Table S2. Among the 69 evaluated responses, 55 (79.7%) were rated as having a low risk of bias, while 14 (20.3%) were considered to have a moderate risk of bias.

### 3.4 Prevalence of poor sleep quality in HD patients

**FIGURE 2:**
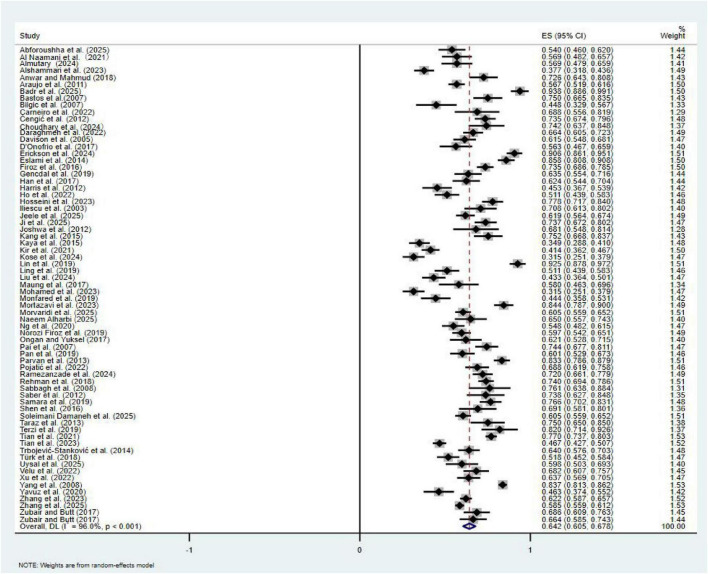
Forest plot of pooled prevalence of poor sleep quality in HD patients.

Owing to the substantial heterogeneity observed across studies, a random-effects model was adopted for the meta-analysis. As shown in Figure 2, the pooled global prevalence of poor sleep quality among HD patients was 64.2% (95% CI: 60.5–67.8%, I^2^ = 96.0%).

### 3.5 Subgroup analysis and meta-regression analysis

Subgroup analysis was conducted to examine the sources of heterogeneity between studies (Table 2). Subgroup analysis based on country income level (Supplementary Figure S1) revealed no significant difference in the pooled prevalence of poor sleep quality between high-income (64.0%, 95% CI: 54.0–73.9%) and low- and middle-income countries (64.2%, 95% CI: 60.3–68.2%). When stratified by study design (Supplementary Figure S2), cross-sectional studies (64.5%, 95% CI: 60.8–68.3%) and cohort studies (56.5%, 95% CI: 33.4–79.6%) demonstrated similar prevalence of poor sleep quality among HD patients. Subgroup analysis by PSQI cut-off value (Supplementary Figure S3) revealed a significant negative correlation (*P* < 0.05). Specifically, the pooled prevalence of poor sleep quality decreased from 70.5% (95% CI: 65.4–75.6%) at a cut-off of ≥ 5–60.8% (95% CI: 56.4–65.2%) at a cut-off of ≥ 6 and then to 53.8% (95% CI: 39.4–68.3%) at a cut-off of ≥ 7.

**TABLE 2:** Subgroup analyses of the prevalence of poor sleep quality among HD patients.

Subgroups	Number of studies	Prevalence (95% CI)	Heterogeneity		*P*-values across subgroups
			I^2^	*P*-values	
Income level High income Low and middle income					0.964
	13	64.0% (54.0–73.9%)	95.2%	*P* < 0.001	
	56	64.2% (60.3–68.2%)	96.2%	*P* < 0.001	
Study design Cross-sectional Cohort					0.502
	66	64.5% (60.8–68.3%)	95.8%	*P* < 0.001	
	3	56.5% (33.4–79.6%)	98.6%	*P* < 0.001	
Cut-off values ≥ 5 ≥ 6 ≥ 7					0.006
	26	70.5% (65.4–75.6%)	94.0%	*P* < 0.001	
	41	60.8% (56.4–65.2%)	95.4%	*P* < 0.001	
	2	53.8% (39.4–68.3%)	92.8%	*P* < 0.001	

HICS, high-income countries; MICS, middle-income countries; AIS, Athens Insomnia Scale; ISI, Insomnia Severity Index.

The results of the univariate meta-regression (Table 3) demonstrated no significant associations between the pooled prevalence of poor sleep quality and the potential moderators, including sample size, mean age, dialysis duration, and female proportion (all *P* > 0.05).

**TABLE 3:** Meta-regression results.

Variables	Number of studies	Coefficient	Standard error	95%CI	*t*–values	*P*-values
Sample size	69	-5.07 × 10^−5^	−8.53 × 10^−5^	−0.0002–0.0001	−0.59	0.554
Mean age (years)	52	0.0023	0.0038	0.0053–0.0099	0.61	0.543
Dialysis duration (years)	20	0.0021	0.0217	−0.0434–0.0476	0.10	0.924
Female (%)	69	−0.0012	0.0024	−0.0059–0.0036	−0.50	0.620

### 3.6 Publication bias and sensitivity analysis

**FIGURE 3:**
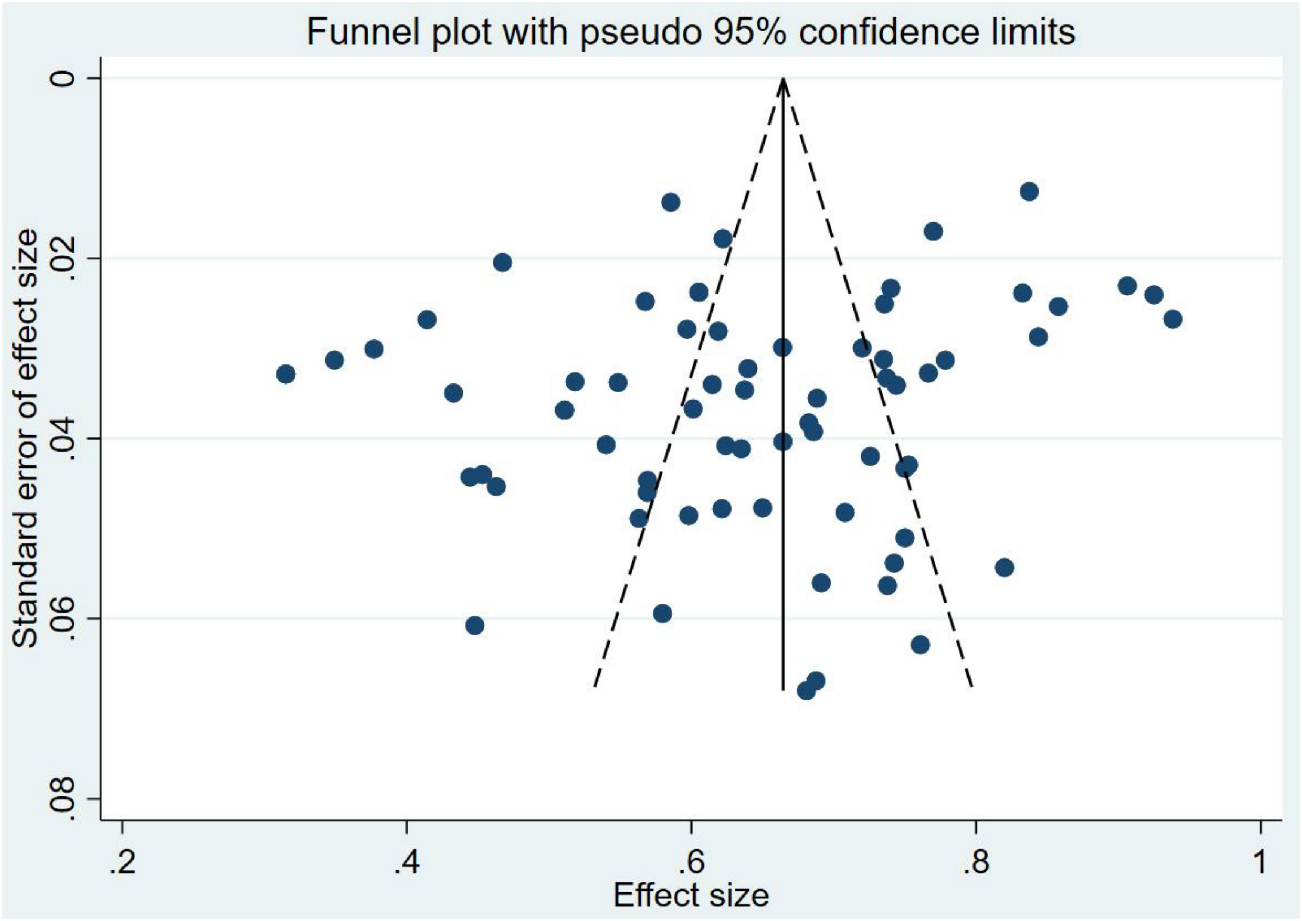
Funnel plot of prevalence of poor sleep quality in HD patients.

Neither visual inspection of the funnel plot (Figure 3) nor statistical evaluation by Egger’s test revealed any evidence of substantial publication bias (Egger’s test: *t* = 15.60, *P* = 0.055, Supplementary Figure S4). Furthermore, the sensitivity analysis confirmed that the pooled prevalence was robust (Supplementary Table S3).

## 4 Discussion

Given that poor sleep quality is a distressing symptom that significantly impairs quality of life in HD patients (8), this study pooled its prevalence in a global sample of 14,998 participants across 19 countries. To the best of our knowledge, this is the first meta-analysis to comprehensively estimate the global prevalence of poor sleep quality in this population. The pooled global prevalence of poor sleep quality among HD patients was 64.2%, which is substantially higher than rates reported in the general population and stroke survivors (77, 78). These findings are in line with expectations, since the various disease and treatment-related issues experienced by HD patients make them susceptible to sleep disturbances (23–26). Firstly, despite regular dialysis, the persistent accumulation of uremic toxins can impair central nervous system function, leading to conditions such as restless legs syndrome and periodic limb movements during sleep, which significantly disrupt sleep initiation in HD patients (24). In addition, the psychological burden of being dependent on dialysis, coupled with concerns about prognosis and socioeconomic issues, usually results in clinical anxiety and depression. These conditions have been demonstrated to be one of the primary contributors to sleep disturbances in these patients (33). Finally, the rigorous daytime schedule of hemodialysis, which generally requires a considerable time commitment several days a week for HD patients, interrupts their normal daily rhythms and is an important cause of sleep disturbances at night (45).

Subgroup analysis results indicated no significant difference in the pooled prevalence of poor sleep quality between high-income (64.0%, 95% CI: 54.0–73.9%) and low- and middle-income countries (64.2%, 95% CI: 60.3–68.2%). These findings may be attributed to several factors. First of all, the mechanisms leading to sleep disturbances in HD patients, such as the accumulation of uremic toxins, electrolyte imbalances, and the comorbidity burden, are inherently connected to renal failure itself (65). Furthermore, the widespread use of the PSQI as a standardized assessment tool ensures consistent measurement of outcomes, thereby reducing potential diagnostic variability that might otherwise result from economic disparities between countries (41–43). Lastly, economic status may be a less relevant indicator than specific factors such as dialysis adequacy, shift work patterns, or the level of family support, all of which have a more direct impact on sleep quality.

In subgroup analyses, the pooled prevalence of poor sleep quality was observed to be similar in both cross-sectional (64.5%, 95% CI: 60.8–68.3%) and cohort studies (56.5%, 95% CI: 33.4–79.6%). In our study, only the prevalence of poor sleep quality at baseline in cohort studies was included in the analysis. At baseline assessment, patients in longitudinal studies are essentially equivalent to those in cross-sectional studies, as they share comparable disease status, treatment exposure, and demographic characteristics before any specific follow-up occurs (79). The factors influencing sleep quality are therefore consistent across both study designs at this point. Therefore, for the purpose of estimating prevalence, information derived from baseline data in high-quality cohort studies should be considered as reliable.

In the stratified analysis based on cut-off values, significant differences were observed in the pooled prevalence of poor sleep quality among studies using different thresholds. Specifically, the pooled prevalence of poor sleep quality decreased from 70.5% (95% CI: 65.4–75.6%) at a cut-off of ≥ 5–60.8% (95% CI: 56.4–65.2%) at a cut-off of ≥ 6 and then to 53.8% (95% CI: 39.4–68.3%) at a cut-off of ≥ 7. Lower cut-off values inherently broaden the scope of case identification, encompassing individuals with milder clinical symptoms. Conversely, applying more stringent thresholds makes the diagnostic criteria for defining poor sleep quality more rigorous, restricting case designation to those exhibiting more pronounced clinical features. These results highlight the fact that choosing cut-off values has a crucial impact on both epidemiological estimates and the potential for developing targeted interventions to address the spectrum of sleep disorder severity in this population.

Given the high prevalence of poor sleep quality in HD patients, it is recommended to integrate sleep assessment into the routine clinical management of HD patients. Specifically, standardized tools should be used for screening during outpatient visits and follow-ups at the clinical screening level. Additionally, clinical nurses can incorporate sleep monitoring into daily assessments and provide sleep hygiene guidance. Finally, multidisciplinary teams integrating psychiatric and psychological services with rehabilitation therapy should be established to develop individualized intervention strategies.

## 5 Limitations

Some limitations should be considered. Firstly, consistent with other meta-analyses conducted on prevalence studies, the included studies exhibited a high level of heterogeneity. However, the heterogeneity remained unexplained by our subgroup and meta-regression analyses. Secondly, despite the lack of a statistical association between prevalence and income level in our subgroup analysis, the overrepresentation of articles from low- and middle-income countries in the included studies limits the robustness of this conclusion for high-income countries. Thirdly, the measurement of poor sleep quality relies on self-reporting tools, which may introduce recall bias. Fourthly, we excluded articles published in any language other than English, which may introduce potential selection bias. Finally, the findings of this study should be interpreted with caution, as they are primarily derived from the pooled results of multiple small-sample observational studies, and the statistical power of the publication bias test is limited.

## 6 Conclusion

In conclusion, this meta-analysis demonstrates that poor sleep quality is a common adverse symptom experienced by HD patients. Given the negative impact of poor sleep quality on HD patients, it is essential to routinely evaluate their sleep quality and implement evidence-based interventions.
