# Supplementary material for: Global prevalence of poor sleep quality in hemodialysis patients: a systematic review and meta-analysis
Source: Front Med (Lausanne). 2026 Feb 23;13:1770352. doi: 10.3389/fmed.2026.1770352 (PMC12967946; doi:10.3389/fmed.2026.1770352)
Supplement: Supplementary file 1 [file Data_Sheet_1.docx]

**Supplemental Materials**

Table S1. The search strategies (Date Run: 20/10/2025)

| Databases | Step | Search Strategies |
| --- | --- | --- |
| PubMed | #1 | "dialysis"[Mesh Terms] Sort by: Best match |
|  | #2 | "dialysis"[Title/Abstract] OR "renal replacement therapy"[Title/Abstract] OR "hemodialysis"[Title/Abstract] OR "haemodialysis"[Title/Abstract] OR "maintenance hemodialysis"[Title/Abstract] OR "maintenance haemodialysis"[Title/Abstract] OR "MHD"[Title/Abstract] Sort by: Best match |
|  | #3 | "sleep quality"[Mesh Terms] Sort by: Best match |
|  | #4 | "sleep quality"[Title/Abstract] OR "sleeping quality"[Title/Abstract] OR "quality of sleep"[Title/Abstract] OR "quality of sleeping"[Title/Abstract] OR  "sleep disorders"[Title/Abstract] OR "[sleep initiation and maintenance disorders](https://www.ncbi.nlm.nih.gov/mesh/68007319)"[Title/Abstract] OR "Pittsburgh sleep quality index"[Title/Abstract] OR "PSQI"[Title/Abstract]  Sort by: Best match |
|  | #5 | #1 OR #2 Sort by: Best match |
|  | #6 | #3 OR #4 Sort by: Best match |
|  | #8 | #5 AND #6 Sort by: Best match |
| Web of Science | #1 | **Ts=(dialysis) OR TS=(**renal replacement therapy) OR TS=(hemodialysis) OR Ts=(haemodialysis) **OR TS=(**maintenance hemodialysis) OR TS=(maintenance hemodialysis) OR TS=(MHD) |
|  | #2 | **Ts=(**sleep quality**) OR Ts=(**sleeping quality) OR **Ts=(quality of sleep) OR TS=(quality of sleeping**) OR TS=(sleep disorders) OR TS=([sleep initiation and maintenance disorders](https://www.ncbi.nlm.nih.gov/mesh/68007319)) OR TS=(Pittsburgh Sleep Quality Index) OR TS=(PSQI) |
|  | #3 | #1 AND #2 |
| Scopus | #1 | TITLE-ABS-KEY ("dialysis" OR "renal replacement therapy" OR "hemodialysis" OR "haemodialysis" OR "maintenance hemodialysis" OR "maintenance haemodialysis" OR "MHD") |
|  | #2 | TITLE-ABS-KEY ("sleep quality" OR "sleeping quality" OR "quality of sleep" OR "quality of sleeping" OR "sleep disorders" OR "[sleep initiation and maintenance disorders](https://www.ncbi.nlm.nih.gov/mesh/68007319)" OR "Pittsburgh sleep quality index" OR "PSQI") |
|  | #3 | #1 AND #2 |
| Embase | **#1** | **'dialysis'/exp** |
|  | **#2** | **'**renal replacement therapy**'/exp** |
|  | **#3** | **'hemodialysis'/exp** |
|  | **#4** | 'dialysis**':ti,ab,kw OR '**renal replacement therapy**':ti,ab,kw OR 'hemodialysis':ti,ab,kw OR 'haemodialysis':ti,ab,kw OR '**maintenance hemodialysis**':ti,ab,kw OR '**maintenance haemodialysis**':ti,ab,kw OR 'MHD':ti,ab,kw** |
|  | **#5** | **'sleep quality'/exp** |
|  | **#6** | 'sleep quality**':ti,ab,kw OR 'sleeping quality':ti,ab,kw OR 'sleep of quality':ti,ab,kw OR 'sleeping of quality':ti,ab,kw OR 'sleep disorders':ti,ab,kw OR '**[sleep initiation and maintenance disorders](https://www.ncbi.nlm.nih.gov/mesh/68007319)**' :ti,ab,kw OR '**Pittsburgh Sleep Quality Index**':ti,ab,kw OR 'PSQI':ti,ab,kw** |
|  | #7 | #1 OR #2 OR #3 OR #4 |
|  | #8 | #5 OR #6 |
|  | #9 | #7 AND #8 |
| Cochrane Library | #1 | MeSH descriptor: [Renal Dialysis] explode all trees |
|  | #2 | (dialysis)**:ti,ab,kw OR (**renal replacement therapy)**:ti,ab,kw OR (hemodialysis):ti,ab,kw OR (haemodialysis):ti,ab,kw OR (**maintenance hemodialysis)**:ti,ab,kw OR (**maintenance haemodialysis)**:ti,ab,kw OR (MHD):ti,ab,kw** |
|  | #3 | MeSH descriptor: [Sleep Quality] explode all trees |
|  | #4 | (sleep quality)**:ti,ab,kw OR (sleeping quality):ti,ab,kw OR (sleep of quality):ti,ab,kw OR (sleeping of quality):ti,ab,kw OR (sleep disorders):ti,ab,kw OR (**[sleep initiation and maintenance disorders](https://www.ncbi.nlm.nih.gov/mesh/68007319))**:ti,ab,kw OR (**Pittsburgh Sleep Quality Index)**:ti,ab,kw OR (PSQI):ti,ab,kw** |
|  | #5 | #1 OR #2 |
|  | #6 | #3 OR #4 |
|  | #7 | #5 AND #6 |
| CINAHL | S1 | **TI dialysis OR TI** renal replacement therapy OR TI hemodialysis OR TI haemodialysis **OR TI** maintenance hemodialysis OR TI maintenance hemodialysis OR TI MHD |
|  | S2 | **TI** sleep quality **OR TI** sleeping quality OR **TI quality of sleep OR TI quality of sleeping** OR TI sleep disorders OR TI [sleep initiation and maintenance disorders](https://www.ncbi.nlm.nih.gov/mesh/68007319)OR TI Pittsburgh Sleep Quality Index OR TI PSQI |
|  | S3 | S1 AND S2 |
| PsycINFO | S1 | **TI dialysis OR TI** renal replacement therapy OR TI hemodialysis OR TI haemodialysis **OR TI** maintenance hemodialysis OR TI maintenance hemodialysis OR TI MHD |
|  | S2 | **TI** sleep quality **OR TI** sleeping quality OR **TI quality of sleep OR TI quality of sleeping** OR TI sleep disorders OR TI [sleep initiation and maintenance disorders](https://www.ncbi.nlm.nih.gov/mesh/68007319)OR TI Pittsburgh Sleep Quality Index OR TI PSQI |
|  | S3 | S1 AND S2 |

Table S2. Quality assessment results of included studies.

| Study | Q1 | Q2 | Q3 | Q4 | Q5 | Q6 | Q7 | Q8 | Q9 | Overall |
| --- | --- | --- | --- | --- | --- | --- | --- | --- | --- | --- |
| Abforoushha et al. (2025) | Y | Y | Y | Y | Y | Y | Y | Y | Y | L |
| Al Naamani et al.（2021） | Y | N | U | Y | Y | Y | Y | Y | Y | L |
| Almutary (2024) | Y | N | Y | Y | Y | Y | Y | Y | Y | L |
| Alshammari et al. (2023) | Y | N | U | Y | U | Y | Y | Y | U | L |
| Anwar and Mahmud (2018) | Y | Y | Y | Y | U | Y | Y | Y | U | L |
| Araujo et al. (2011) | Y | Y | Y | Y | U | Y | Y | Y | U | L |
| Badr et al. (2025) | N | N | Y | Y | Y | Y | Y | Y | Y | L |
| Bastos et al.(2007) | N | N | U | Y | Y | Y | Y | Y | U | L |
| Bilgic et al. (2007) | N | N | U | Y | U | Y | Y | Y | U | M |
| Carneiro et al. (2022) | N | N | U | Y | U | Y | Y | Y | U | M |
| Čengić et al. (2012) | Y | N | U | Y | Y | Y | Y | Y | U | L |
| Choudhary et al. (2024） | N | N | N | Y | U | Y | Y | Y | U | M |
| Daraghmeh et al. (2022) | Y | N | Y | Y | Y | Y | Y | Y | Y | L |
| Davison et al. (2005) | Y | N | Y | Y | Y | Y | Y | Y | Y | L |
| D'Onofrio et al. (2017) | Y | N | U | Y | Y | Y | Y | Y | Y | L |
| Erickson et al. (2024) | Y | N | U | Y | Y | Y | Y | Y | U | L |
| Eslami et al. (2014) | Y | N | Y | Y | Y | Y | Y | Y | U | L |
| Firoz et al. (2016) | Y | N | N | Y | Y | Y | Y | Y | U | L |
| Gencdal et al. (2019) | Y | N | N | Y | Y | Y | Y | Y | U | L |
| Han et al. (2017) | Y | Y | N | Y | Y | Y | Y | Y | Y | L |
| Harris et al. (2012) | Y | U | U | Y | U | Y | Y | Y | U | L |
| Ho et al. (2022) | Y | Y | U | Y | Y | Y | Y | Y | U | L |
| Hosseini et al. (2023) | Y | N | U | Y | Y | Y | Y | Y | U | L |
| Iliescu et al. (2003) | N | N | N | Y | U | Y | Y | Y | N | M |
| Jeele et al. (2025) | Y | N | Y | Y | Y | Y | Y | Y | Y | L |
| Ji et al. (2025) | Y | N | Y | Y | Y | Y | Y | Y | N | L |
| Joshwa et al. (2012) | N | N | U | Y | U | Y | Y | Y | U | M |
| Kang et al. (2015) | Y | N | Y | Y | U | Y | Y | Y | U | L |
| Kaya et al. (2015) | Y | N | U | Y | Y | Y | Y | Y | U | L |
| Kir et al. (2021) | Y | N | Y | Y | Y | Y | Y | Y | U | L |
| Kose et al. (2024) | Y | U | Y | Y | Y | Y | Y | Y | U | L |
| Lin et al. (2019) | Y | N | Y | Y | Y | Y | Y | Y | U | L |
| Ling et al. (2019) | Y | N | Y | Y | Y | Y | Y | Y | Y | L |
| Liu et al. (2024) | Y | U | Y | Y | U | Y | Y | Y | U | L |
| Maung et al. (2017) | N | N | N | Y | U | Y | Y | Y | U | M |
| Mohamed et al. (2023) | Y | N | U | Y | Y | Y | Y | Y | U | L |
| Monfared et al. (2019) | N | N | U | Y | U | Y | Y | Y | U | M |
| Mortazavi et al. (2023) | Y | N | Y | Y | Y | Y | Y | Y | U | L |
| Morvaridi et al. (2025) | Y | N | Y | Y | Y | Y | Y | Y | U | L |
| Naeem Alharbi (2025) | N | N | U | Y | U | Y | Y | Y | U | M |
| Ng et al. (2020) | Y | U | Y | Y | Y | Y | Y | Y | Y | L |
| Norozi Firoz et al. (2019) | Y | N | Y | Y | Y | Y | Y | Y | U | L |
| Ongan and Yuksel (2017) | N | U | N | Y | Y | Y | Y | Y | U | L |
| Pai et al. (2007) | Y | N | U | Y | Y | Y | Y | Y | U | L |
| Pan et al. (2019) | Y | N | Y | Y | Y | Y | Y | Y | U | L |
| Parvan et al. (2013) | Y | N | Y | Y | Y | Y | Y | Y | U | L |
| Pojatić et al. (2022) | Y | Y | U | Y | Y | Y | Y | Y | Y | L |
| Ramezanzade et al. (2024) | Y | N | U | Y | Y | Y | Y | Y | U | L |
| Rehman et al. (2018) | Y | N | Y | Y | Y | Y | Y | Y | U | L |
| Sabbagh et al. (2008) | N | N | N | Y | U | Y | Y | Y | U | M |
| Saber et al. (2012) | N | N | N | Y | U | Y | Y | Y | U | M |
| Samara et al. (2019) | Y | N | Y | Y | Y | Y | Y | Y | Y | L |
| Shen et al. (2016) | N | N | N | Y | U | Y | Y | Y | U | M |
| Soleimani Damaneh et al. (2025) | Y | U | Y | Y | Y | Y | Y | Y | N | L |
| Taraz et al. (2013) | N | N | N | Y | U | Y | Y | Y | U | M |
| Terzi et al. (2019) | N | N | N | Y | U | Y | Y | Y | U | M |
| Tian et al. (2021) | Y | N | U | Y | Y | Y | Y | Y | U | L |
| Tian et al. (2023) | Y | N | U | Y | Y | Y | Y | Y | U | L |
| Trbojević-Stanković et al. (2014) | Y | N | U | Y | Y | Y | Y | Y | U | L |
| Türk et al. (2018) | Y | N | U | Y | Y | Y | Y | Y | U | L |
| Uysal et al. (2025) | N | N | U | Y | U | Y | Y | Y | U | M |
| Velu et al. (2022) | Y | N | Y | Y | Y | Y | Y | Y | U | L |
| Xu et al. (2022) | Y | N | Y | Y | Y | Y | Y | Y | U | L |
| Yang et al. (2008) | Y | N | Y | Y | Y | Y | Y | Y | U | L |
| Yavuz et al. (2020) | Y | N | U | Y | Y | Y | Y | Y | U | L |
| Zhang et al. (2023) | Y | N | Y | Y | Y | Y | Y | Y | U | L |
| Zhang et al. (2025) | Y | N | Y | Y | Y | Y | Y | Y | Y | L |
| Zubair and Butt (2017) | Y | N | Y | Y | Y | Y | Y | Y | U | L |
| Zubair and Butt (2017) | Y | N | Y | Y | Y | Y | Y | Y | U | L |

Notes: Y: Yes, N: No, U: Unclear. L: Low risk, M: Moderate risk, H: High risk.

Q1: Was the sample frame appropriate to address the target population?

Q2: Were study participants sampled in an appropriate way?

Q3: Was the sample size adequate?

Q4: Were the study subjects and the setting described in detail?

Q5: Was the data analysis conducted with sufficient coverage of the identified sample?

Q6: Were valid methods used for the identification of the condition?

Q7: Was the condition measured in a standard, reliable way for all participants?

Q8: Was there appropriate statistical analysis?

Q9: Was the response rate adequate, and if not, was the low response rate managed appropriately?

Each study was categorized by risk of bias as follows: high risk if ≤ 49% of items are responded to with "yes;" moderate risk if 50%-69% of items are responded to with "yes; "and low risk if ≥ 70% of items are responded to with "yes."


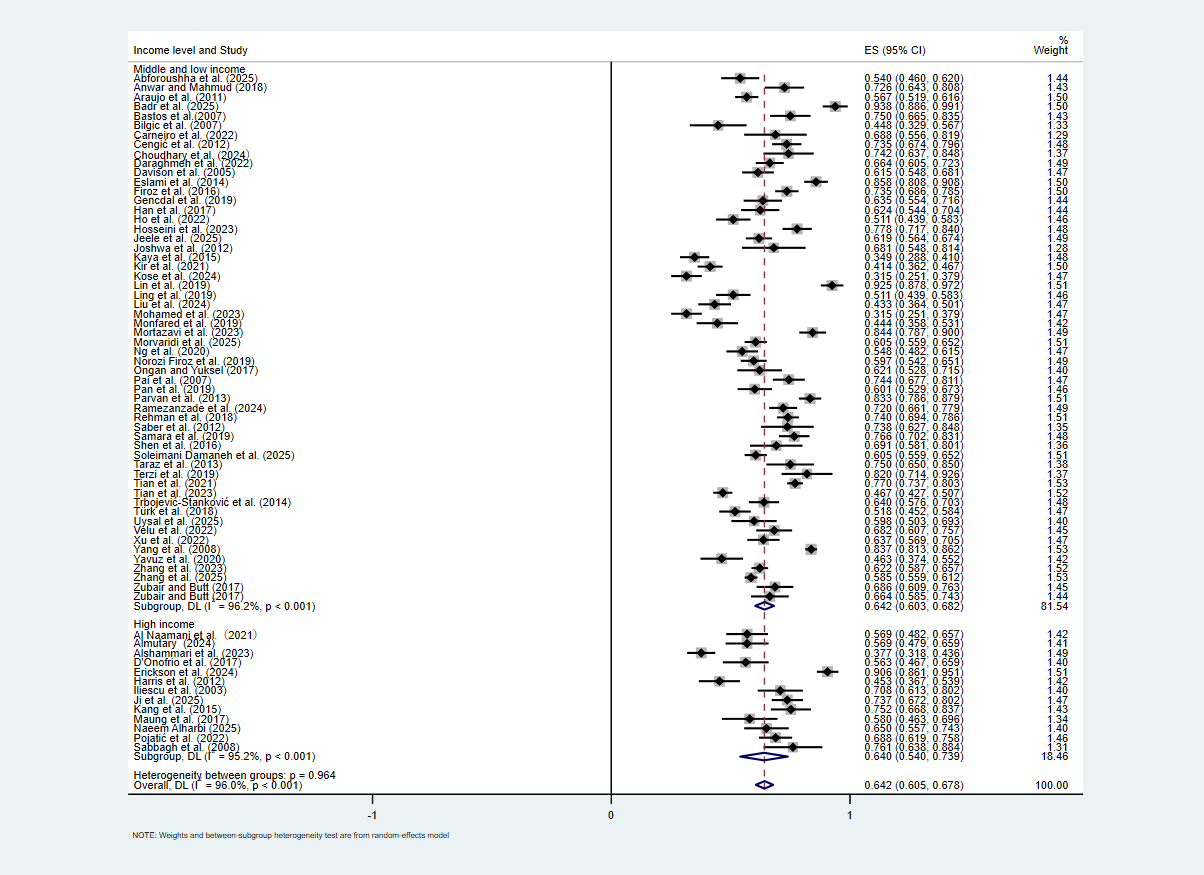


Figure S1. The pooled prevalence of poor sleep quality in HD patients based on income level.


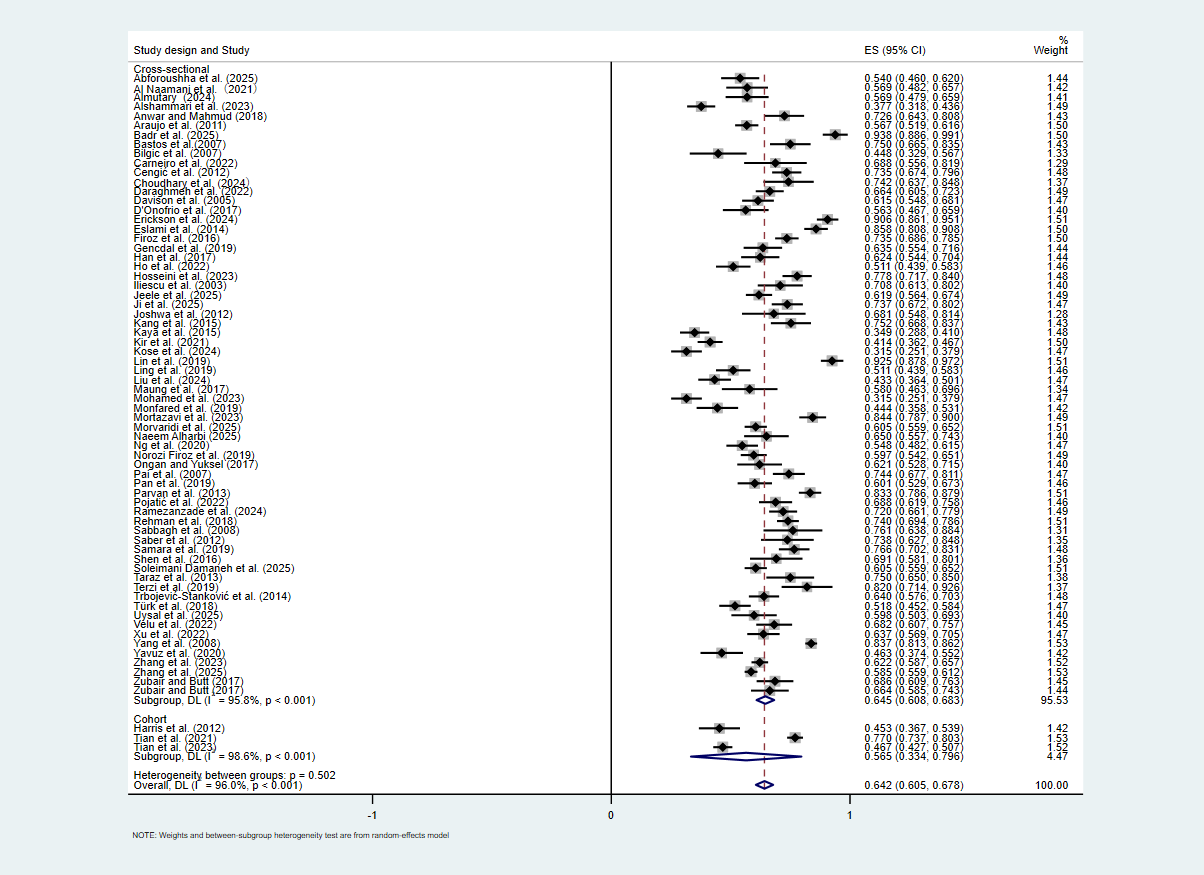


Figure S2. The pooled prevalence of poor sleep quality in HD patients based on study design.


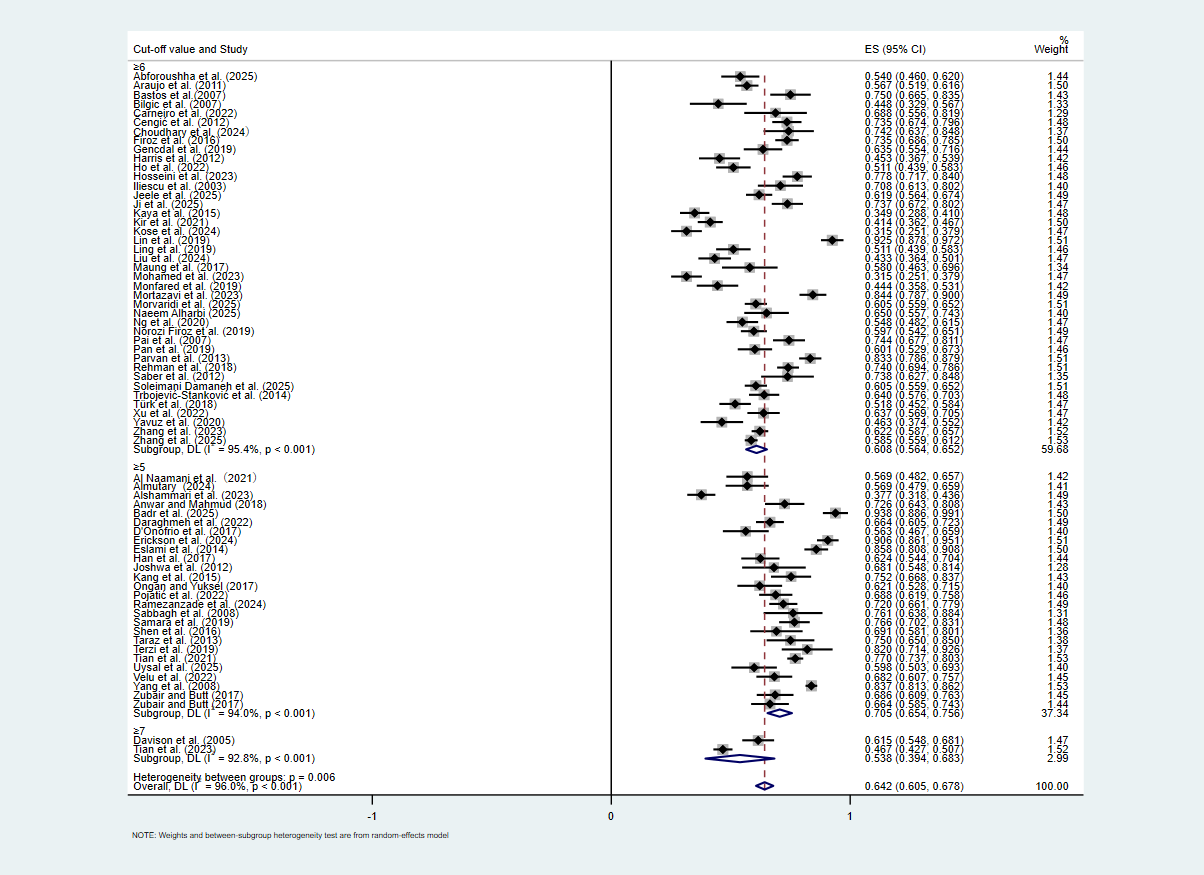


Figure S3. The pooled prevalence of poor sleep quality in HD patients based on cut-off values.


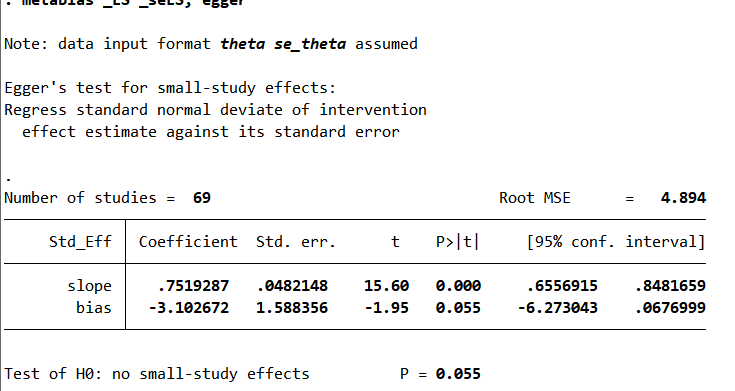


Figure S4. Egger's test results.

Table S3. The results of the sensitivity analysis.

| Omitted Study | Prevalence | 95% CI |
| --- | --- | --- |
| Abforoushha et al. (2025) | 0.643 | 0.607 - 0.680 |
| Al Naamani et al. (2021) | 0.643 | 0.606 - 0.680 |
| Almutary (2024) | 0.643 | 0.606 - 0.680 |
| Alshammari et al. (2023) | 0.646 | 0.610 - 0.682 |
| Anwar and Mahmud (2018) | 0.641 | 0.604 - 0.677 |
| Araujo et al. (2011) | 0.643 | 0.606 - 0.680 |
| Badr et al. (2025) | 0.637 | 0.601 - 0.673 |
| Bastos et al. (2007) | 0.640 | 0.603 - 0.677 |
| Bilgic et al. (2007) | 0.644 | 0.608 - 0.681 |
| Carneiro et al. (2022) | 0.641 | 0.604 - 0.678 |
| Čengić et al. (2012) | 0.640 | 0.604 - 0.677 |
| Choudhary et al. (2024) | 0.640 | 0.604 - 0.677 |
| Daraghmeh et al. (2022) | 0.641 | 0.604 - 0.678 |
| Davison et al. (2005) | 0.642 | 0.605 - 0.679 |
| D'Onofrio et al. (2017) | 0.643 | 0.606 - 0.680 |
| Erickson et al. (2024) | 0.638 | 0.602 - 0.674 |
| Eslami et al. (2014) | 0.639 | 0.602 - 0.675 |
| Firoz et al. (2016) | 0.640 | 0.603 - 0.677 |
| Gencdal et al. (2019) | 0.642 | 0.605 - 0.679 |
| Han et al. (2017) | 0.642 | 0.605 - 0.679 |
| Harris et al. (2012) | 0.645 | 0.608 - 0.681 |
| Ho et al. (2022) | 0.644 | 0.607 - 0.680 |
| Hosseini et al. (2023) | 0.640 | 0.603 - 0.677 |
| Iliescu et al. (2003) | 0.641 | 0.604 - 0.678 |
| Jeele et al. (2025) | 0.642 | 0.605 - 0.679 |
| Ji et al. (2025) | 0.640 | 0.604 - 0.677 |
| Joshwa et al. (2012) | 0.641 | 0.605 - 0.678 |
| Kang et al. (2015) | 0.640 | 0.603 - 0.677 |
| Kaya et al. (2015) | 0.646 | 0.610 - 0.682 |
| Kir et al. (2021) | 0.645 | 0.609 - 0.681 |
| Kose et al. (2024) | 0.647 | 0.611 - 0.682 |
| Lin et al. (2019) | 0.637 | 0.602 - 0.673 |
| Ling et al. (2019) | 0.644 | 0.607 - 0.680 |
| Liu et al. (2024) | 0.645 | 0.608 - 0.681 |
| Maung et al. (2017) | 0.643 | 0.606 - 0.679 |
| Mohamed et al. (2023) | 0.647 | 0.611 - 0.682 |
| Monfared et al. (2019) | 0.645 | 0.608 - 0.681 |
| Mortazavi et al. (2023) | 0.639 | 0.602 - 0.675 |
| Morvaridi et al. (2025) | 0.642 | 0.605 - 0.679 |
| Naeem Alharbi (2025) | 0.642 | 0.605 - 0.679 |
| Ng et al. (2020) | 0.643 | 0.606 - 0.680 |
| Norozi Firoz et al. (2019) | 0.642 | 0.606 - 0.679 |
| Ongan and Yuksel (2017) | 0.642 | 0.605 - 0.679 |
| Pai et al. (2007) | 0.640 | 0.603 - 0.677 |
| Pan et al. (2019) | 0.642 | 0.606 - 0.679 |
| Parvan et al. (2013) | 0.639 | 0.602 - 0.676 |
| Pojatić et al. (2022) | 0.641 | 0.604 - 0.678 |
| Ramezanzade et al. (2024) | 0.641 | 0.604 - 0.678 |
| Rehman et al. (2018) | 0.640 | 0.603 - 0.677 |
| Sabbagh et al. (2008) | 0.640 | 0.604 - 0.677 |
| Saber et al. (2012) | 0.641 | 0.604 - 0.677 |
| Samara et al. (2019) | 0.640 | 0.603 - 0.677 |
| Shen et al. (2016) | 0.641 | 0.604 - 0.678 |
| Soleimani Damaneh et al. (2025) | 0.642 | 0.605 - 0.679 |
| Taraz et al. (2013) | 0.640 | 0.604 - 0.677 |
| Terzi et al. (2019) | 0.639 | 0.603 - 0.676 |
| Tian et al. (2021) | 0.640 | 0.603 - 0.677 |
| Tian et al. (2023) | 0.645 | 0.608 - 0.681 |
| Trbojević-Stanković et al. (2014) | 0.642 | 0.605 - 0.679 |
| Türk et al. (2018) | 0.644 | 0.607 - 0.680 |
| Uysal et al. (2025) | 0.642 | 0.606 - 0.679 |
| Velu et al. (2022) | 0.641 | 0.604 - 0.678 |
| Xu et al. (2022) | 0.642 | 0.605 - 0.679 |
| Yang et al. (2008) | 0.639 | 0.603 - 0.675 |
| Yavuz et al. (2020) | 0.644 | 0.608 - 0.681 |
| Zhang et al. (2023) | 0.642 | 0.605 - 0.680 |
| Zhang et al. (2025) | 0.643 | 0.605 - 0.680 |
| Zubair and Butt (2017) - First Entry | 0.641 | 0.604 - 0.678 |
| Zubair and Butt (2017) - Second Entry | 0.641 | 0.605 - 0.678 |
